# Outcomes of septic shock due to multidrug resistant bacteraemia treated with phosphomycine

**DOI:** 10.1186/2197-425X-3-S1-A87

**Published:** 2015-10-01

**Authors:** A Theohari, E Koutsioumpa, E Manoulakas, K Mantzarlis, D Makris, E Zakynthinos

**Affiliations:** University Hospital of Larisa, ICU, Larisa, Greece

## Introduction

The management of septic shock due to gram negative infections is challenging due to the multidrug antibiotic resistance of some bacterial species.

## Objectives

We investigated outcomes of critical care patients with septic shock due to gram negative bacteraimia susceptible only to phosphomycine.

## Methods

Data from septic shock cases hospitalized in a tertiary ICU, during 2014, were retrospectively collected if patients had received phosphomycine i.v. at least for seventy-two hours, as rescue therapy for septic shock bacteraemia due to multidrug resistant (including colistin resistance) bacteria. Main outcomes (i.e. death in ICU, recovery from septic shock) were compared with cases of septic shock due to multidrug resistant (but susceptible at least to colistin) gram negative bacteraemia (first ICU episode) who received appropriate antibiotic therapy for at least seventy-two hours (Controls).

## Results

Twelve Cases and 14 Controls were included in the study. Median(IQR) age (years) were 56(45.5-69.2) and 62(57-65), APACHE II 21(20-31) and 18(14-24.5), ICU day of septic shock 63.5(39.5-129.7) and 10(5-16). Duration (days) of phosphomycine treatment was 12.5(9.7-15). Initial empirical antibiotic therapy was appropriate in 62.5% of Controls. Four (33%) Case and 9 (64%) Controls recovered from septic shock in both groups (p = 0.23). ICU duration in Cases and Controls were 73(41-152) and 25(16-33) (p = 0.02) while overall mortality was 75% and 67% respectively (p > 0.5).

## Conclusions

The use of phosphomycine as rescue therapy in septic shock due to multidrug resistant bacteraemia resulted in low recovery rates although not significantly different compared to patients with septic shock due to multidrug resistant (susceptible to more than two antibiotics including colistin) bacteria.

